# An epigenetically inherited UV hyper-resistance phenotype in *Saccharomyces cerevisiae*

**DOI:** 10.1186/s13072-022-00464-5

**Published:** 2022-08-20

**Authors:** Rachel M. Reardon, Amanda K. Walsh, Clairine I. Larsen, LauraAnn H. Schmidberger, Lillian A. Morrow, Adriane E. Thompson, Isabel M. Wellik, Jeffrey S. Thompson

**Affiliations:** 1 Department of Biology, Denison University, 213 Talbot Hall, 100 W. College St., Granville, OH 43023 USA; 2 Harvard Medical School, Boston, MA 02115 USA; 3 The Ohio State University College of Medicine, Columbus, OH 43210 USA; 4 Institute for Molecular Virology and McArdle Laboratory for Cancer Research, University of Wisconsin-Madison, Madison, WI 53705 USA; 5 Department of Biochemistry and Biophysics, University of California San Francisco, San Francisco, CA 94143 USA; 6 Scripps Research Institute, La Jolla, CA 92037 USA; 7 Grinnell College, Grinnell, IA 50112 USA; 8 Department of Human Genetics, The University of Michigan, Ann Arbor, MI 48109 USA

**Keywords:** Epigenetics, UV, DNA damage, Histone acetylation, Histone methylation, Cell wall, Cell size, Yeast

## Abstract

**Background:**

Epigenetics refers to inheritable phenotypic changes that occur in the absence of genetic alteration. Such adaptations can provide phenotypic plasticity in reaction to environmental cues. While prior studies suggest that epigenetics plays a role in the response to DNA damage, no direct demonstration of epigenetically inheritable processes have been described in this context.

**Results:**

Here we report the identification of an epigenetic response to ultraviolet (UV) radiation in the baker’s yeast *Saccharomyces cerevisiae*. Cells that have been previously exposed to a low dosage of UV exhibit dramatically increased survival following subsequent UV exposure, which we refer to as UV hyper-resistance (UVHR). This phenotypic change persists for multiple mitotic generations, without any indication of an underlying genetic basis. Pre-exposed cells experience a notable reduction in the amount of DNA damage caused by the secondary UV exposure. While the mechanism for the protection is not fully characterized, our results suggest that UV-induced cell size increases and/or cell wall changes are contributing factors. In addition, we have identified two histone modifications, H3K56 acetylation and H3K4 methylation, that are important for UVHR, potentially serving as mediators of UV protective gene expression patterns, as well as epigenetic marks to propagate the phenotype across cell generations.

**Conclusions:**

Exposure to UV radiation triggers an epigenetically inheritable protective response in baker’s yeast that increases the likelihood of survival in response to subsequent UV exposures. These studies provide the first demonstration of an epigenetically inheritable dimension of the cellular response to DNA damage.

**Supplementary Information:**

The online version contains supplementary material available at 10.1186/s13072-022-00464-5.

## Introduction

The regulation of gene expression plays an essential role in dictating how organisms respond to their environment, resulting in transient phenotypic changes. Regulatable genes react to molecular cues that influence expression patterns via transcription factor function, chromatin structure changes, and other regulatory features. Induced expression patterns typically return to a default state once the triggers are no longer present. In some cases, genes may continue to display persistent modified expression patterns, or they may establish a transcriptionally “poised” state to enable a hyper-reactionary response to subsequent regulatory cues. Furthermore, these persistent expression states may be heritable in parallel with the genetic instructions [1]. In this way, cells can pass on a working memory of gene expression patterns to their daughter cells, driven by the environmental experiences of the parent cell [2]. Such phenomena are commonly referred to as “epigenetic”, implying the inheritance of a supplementary layer of gene expression and phenotypic information.

Epigenetic inheritance is achieved by the establishment and maintenance of reversible DNA-associated modifications [3]. These modifications serve as regulators of gene expression, with potential for mitotic and/or meiotic inheritance. Several distinct types of epigenetic modifications have been identified. One well-studied example is DNA methylation, specifically of cytosine residues in the context of CpG islands, which can silence gene expression through the recruitment of repressive methyl-specific CpG binding proteins [4]. DNA methylation patterns are inheritable through the action of maintenance methyltransferases that coordinate methylation with DNA replication. Histone post-translational modifications serve as another class of epigenetic regulators. Histone proteins, the building blocks of chromatin, are subject to a wide array of modifications, including acetylation, methylation, and phosphorylation [5]. Histone modifications influence gene expression and other DNA-related processes via their impact on chromatin accessibility. While the underlying mechanism of the inheritance of histone modifications and chromatin states remains unresolved, the persistence of locus-specific histone modification patterns across cell generations suggests that these modifications are inheritable to some degree. Finally, non-coding RNAs have also been implicated in epigenetic inheritance [6]. While technically not “modifications”, these RNAs associate with DNA and/or chromatin, whereupon they exert influences on gene expression, such as the X chromosome inactivating Xist RNA in mammals [7]. Many non-coding regulatory RNAs have been identified, some possessing potential epigenetic roles in gene regulation [8].

One specific branch of cell biology where epigenetics may play a role is in the context of the response to DNA damage. DNA is subject to numerous forms of damage, including single- and double-stranded breaks, crosslinks, bulky adduct formation, and nucleotide base alterations/loss [9]. One common source of environmentally derived DNA damage is ultraviolet (UV) radiation, which causes cyclopyrimidine dimers (CPDs), and at high dosages, double-stranded breaks [10]. A wide range of response and repair processes are induced by DNA damage, and various epigenetic modifications have been implicated in DNA damage responses. Specifically, numerous histone modifications have been identified that are important for the response to DNA damage [11], including the extensively studied histone H2AX phosphorylation, which is found in the context of double-stranded breaks. Histone modifications influence chromatin structure, leading to damage-induced changes in gene expression, as well as playing roles in the accessibility of chromatin in the context of DNA repair processes [12].

Despite these modifications being involved in DNA damage response, it has not been established that DNA damage invokes actual epigenetic processes. It is conceivable that a DNA damage event might establish an epigenetically inheritable expression state that serves to alter how the cell and/or its descendants respond to subsequent DNA damage events. Virtually no prior work has been published to directly address this question, although a few studies suggest the existence of such a phenomenon. In one prior investigation, *S. cerevisiae* received an initial UV exposure, followed by a brief recovery period and then a subsequent UV exposure [13]. Pre-exposed cells displayed enhanced CPD removal relative to the unexposed controls. However, the short timing between the two UV exposures raises the possibility that the phenotypic difference is simply due to active expression of DNA repair genes. Furthermore, the brevity of the recovery period precluded the examination of inherited phenotypic change in subsequent cell generations. Other studies have similarly explored the effect of repeated exposures to UV [14, 15], but none of these studies were explicitly designed to address the potential role of epigenetic processes. Thus, the question of whether inheritable epigenetics plays a role in the response to DNA damage is largely unexplored.

This study seeks to investigate questions pertaining to a potential role of inheritable epigenetics in the response to DNA damage, specifically exploring the impact of UV radiation on baker’s yeast, *Saccharomyces cerevisiae*. First, does UV exposure have a phenotypic effect on *S. cerevisiae* that influences the response to subsequent DNA damage events, and is that phenotype inheritable across cell generations? If so, what is the underlying cause of the phenotype? And finally, how is this phenotype inherited across cell generations? As we will present below, we have identified a novel, epigenetically inheritable UV hyper-resistance (UVHR) phenotype, whereby cells that experience a mildly damaging UV exposure event display enhanced survival in response to subsequent UV exposure. This phenotypic change is inherited across multiple mitotic cell generations in a non-genetic manner, involving cellular changes that provide protection against UV-induced DNA damage. We will additionally demonstrate the importance of two key histone modifications that are important for this phenotype, which may serve as inducers for the UV protective changes as well as the form of epigenetically inherited instructions.

## Results

### Pre-exposure to UV radiation enhances cell survival following a secondary UV exposure

**Fig. 1:**
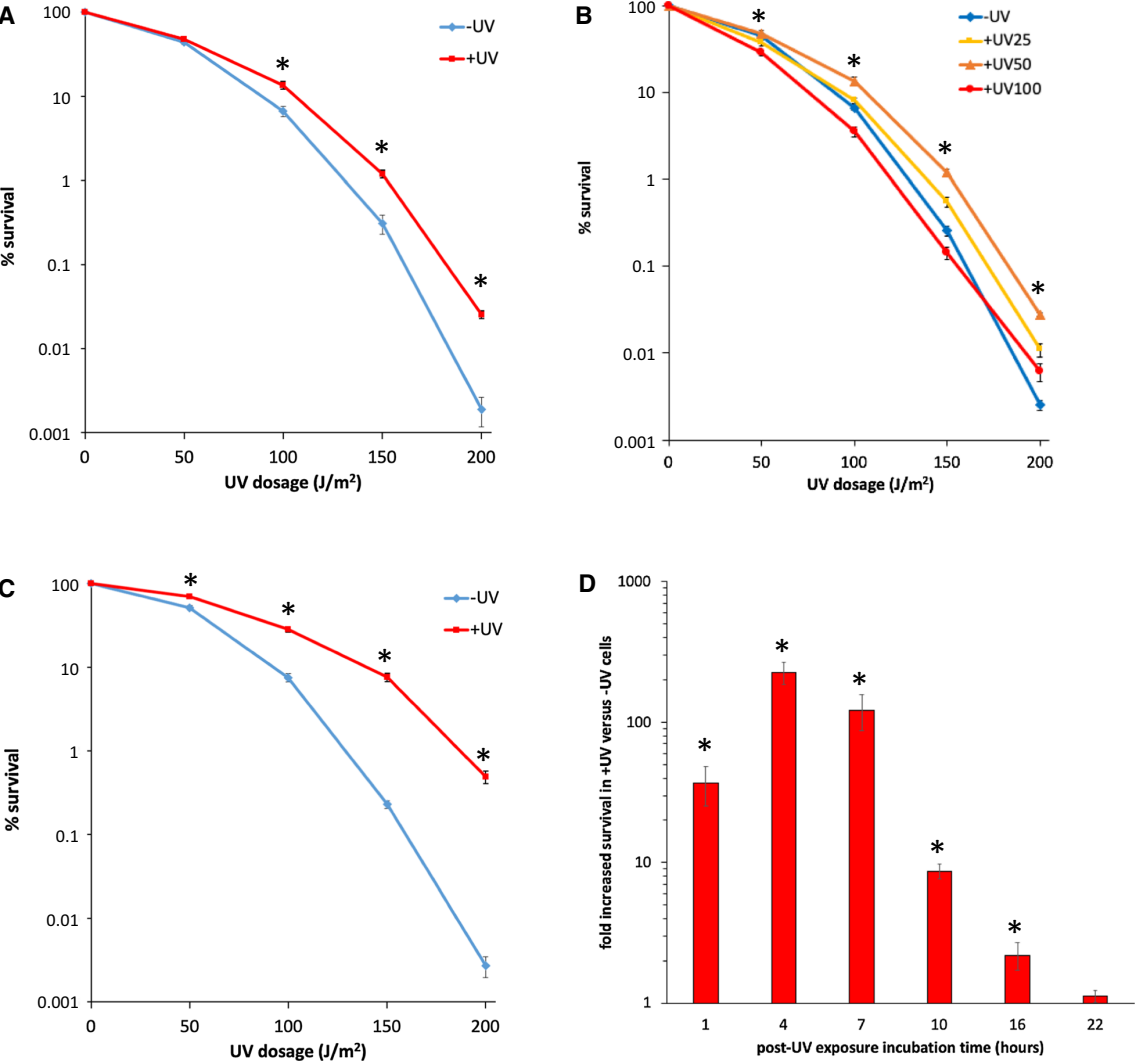
UV exposure induces hyper-resistance to subsequent UV exposure (UVHR). Quantitative UV double-exposure survival assays were done on log-phase yeast cultures (strain BY4741) using a Philips 30 W G30T8 UV lamp at 254 nm. Cultures were serially diluted and plated in duplicate on YEPD agar, exposed to UV, and then incubated in the dark for ~ 6 days. Colonies were counted and used to calculate relative survival frequencies. Assays were done a minimum of three times, with mean ± 1 SE reported for each condition (*p < 0.01). **A**. Yeast cells were exposed to two sequential rounds of UV radiation, first on suspended cells at 50 J/m^2^ (+ UV), along with a mock-exposed control (−UV), followed by a 1 h incubation at 30 °C, and then plating and UV exposure to varying secondary dosages (as indicated on the X axis). **B**. Same as **A**, except with varying initial exposure dosages (25, 50, and 100 J/m^2^, respectively). *, statistically significant differences found at the indicated UV secondary dosages (*p* < 0.01 for all pairwise comparisons, with the following exceptions: at 50 J/m^2^, only + UV50 versus + UV100 and −UV versus + UV100 differences are significant; for all other comparisons, *p* > 0.05; at 100 J/m^2^, −UV versus + UV25, *p* > 0.05; at 150 and 200 J/m^2^, −UV versus + UV100, *p* = 0.03; at 200 J/m^2^, + UV25 versus + UV50 and + UV100, *p* > 0.05). **C**. Same as **A**, except with a 4 h incubation period. **D**. Same as **A**, except with varying incubation times, as indicated on X axis. The graph displays fold difference in survival (+ UV relative to −UV) based on secondary exposure survival at 200 J/m^2^. Values for 1- and 4-h incubation assays are derived from a and c. Values for the 7-, 10-, 16-, and 22-h incubations are derived from assays reported in Additional file 1: Figure S1

We initially sought to determine if exposure to UV radiation affects cell survival following a subsequent UV exposure. A double-exposure protocol was developed, in which log phase yeast cells were split into two groups: a “ + UV” experimental group that received a 50 J/m^2^ exposure to UV radiation; and an unexposed “−UV” control group. Both groups were subsequently incubated for 1 h to allow for repair of DNA damage, and then cells were subjected to secondary UV exposures of varying dosages. Survival frequencies were determined by colony formation on agar plates. We observed that cells which experienced the pre-exposure (+ UV) displayed significantly higher survival rates than control cells following secondary exposures at most dosages (Fig. 1a), ranging from ~ twofold to 11-fold, depending on the secondary exposure dosage. These results indicate that a prior exposure to UV radiation triggers cellular changes that enable enhanced survival in response to subsequent UV exposures. We refer to this as a “UV hyper-resistance” phenotype (UVHR).

To further evaluate this response, we varied the intensity of the initial UV exposure to determine how UVHR is influenced by UV dosage. The same double-exposure protocol was followed, this time with initial exposure intensities of either 25 or 100 J/m^2^. A pre-exposure of 25 J/m^2^ resulted in significant increases in cell survival relative to unexposed cells, although the degree of enhanced survival was reduced relative to what had been observed with the 50 J/m^2^ pre-exposures. Significant increases in survival were observed only at secondary exposures of 150 and 200 J/m^2^, and the fold increase in survival was reduced at all secondary exposure intensities relative to the 50 J/m^2^ pre-exposure group at the same intensities (twofold and fourfold, respectively; Fig. 1b). In contrast, pre-exposure at 100 J/m^2^ led to decreases in survival for the + UV group relative to the −UV group for secondary exposures of 50, 100, and 150 J/m^2^, reversing the trend observed in the other two pre-exposure intensities. + UV cell survival dropped to ~ 0.5-fold relative to −UV cells at these dosages. While cell survival was statistically higher for pre-exposed cells that experienced a 200 J/m^2^ secondary exposure, this fold increase (2.5-fold) was smaller than the difference observed in the other pre-exposure intensity groups. Thus, UVHR is dependent on the intensity of the initial exposure, with a maximal effect observed at 50 J/m^2^.

### UVHR is epigenetically inherited during mitosis

We sought to determine whether UVHR is a persistent and inheritable phenotype. To do so, we extended the incubation time between UV pre-exposure and secondary exposure to provide cells sufficient time to repair UV-induced damage and undergo subsequent rounds of mitotic reproduction before the secondary exposure. Using a 4 h incubation, allowing for ~ 1–2 mitotic cycles (based on observed ~ threefold increases in + UV cell culture density during the incubation period), we observed significant increases in survival of the + UV cells at all secondary exposure dosages, with larger fold increases in survival of + UV cells relative to −UV cells at each dosage compared to the original 1 h incubation (Fig. 1c). Under these conditions, we observed a ~ 200-fold increase in survival in the + UV group at the 200 J/m^2^ secondary exposure. UVHR was observed in cultures that were incubated up to 16 h following the initial exposure (Fig. 1d, Additional file 1: Figure S1). Fold increased survival in + UV cells relative to −UV cells gradually diminished with extended incubation time, reaching a low of twofold after 16 h (at 200 J/m^2^ secondary exposure), and completely disappearing at 22 h. Over a period of 16 h, it is estimated that the initial UV exposed cells have undergone ~ 7–8 rounds of cell division (based on observed ~ 100-fold to 200-fold increases in + UV cell culture density during the incubation period), indicating that the UVHR phenotype is inherited mitotically.

**Fig. 2:**
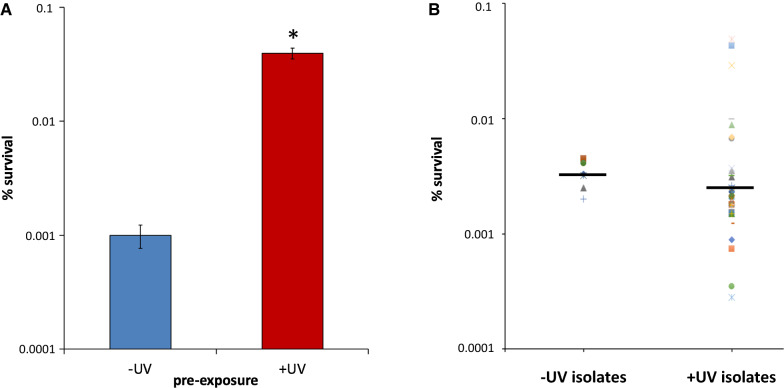
UVHR is epigenetically inherited. UV survival assays were done as described in Fig. 1, with variations as indicated below. **A** Mother cell-free population. Cells were labeled with biotin immediately after the initial UV exposure at 50 J/m^2^ (+ UV), or after mock exposure (−UV). After the 4 h incubation, labeled mother cells were removed from the population with streptavidin-linked magnetic beads. The remaining unlabeled daughter cells were subsequently diluted, plated, and exposed to UV at 200 J/m^2^. *p < 0.01. **B** Isolated colonies from a single UV exposure at 50 J/m^2^, followed by a 4 h incubation. Thirty isolates from UV exposed set (+ UV) and 10 isolates from mock-exposed controls (−UV) were restreaked and incubated for ~ 3 days, and then subsequently cultured, diluted, plated, and exposed to UV (200 J/m^2^). Each isolate was tested twice, and values are reported as means. Horizontal bars indicate the geometric mean for each set of isolates. *p* > 0.1

To further examine the inheritability of the UVHR phenotype, we considered the possibility that UVHR might be exclusively present in the pre-exposed mother cells, which, because of the budding mechanism for reproduction in *S. cerevisiae*, remain part of the secondarily exposed cell population (in gradually decreasing relative numbers). To determine if UVHR was specifically passed onto daughter cells, we used a labeling technique to remove mother cells from the population prior to the secondary exposure. Immediately following UV exposure, mother cells (in both the −UV and + UV cultures) were labeled with biotin, which covalently attaches to the cell wall [16]. Cells were then incubated for 4 h to allow for expansion of the cell populations. Yeast cell walls are synthesized de novo at the site of bud formation [16], thus the biotin label is exclusively retained by the mother cell, and not passed onto the daughter cell. After the incubation period, streptavidin-linked magnetic beads were added to cultures to extract the biotin-labeled mother cells from the population. The remaining daughter cells were then plated and exposed to the secondary UV dosage (200 J/m^2^) and evaluated for viability via colony formation. We observed that the pre-exposed daughter cells (+ UV) exhibited significantly increased survival following the second UV exposure relative to the unexposed (−UV) control population, with a 40-fold increase in survival (Fig. 2a). While the magnitude of the UVHR phenotype was reduced relative to what was previously described above in the unsorted populations under equivalent UV intensity/incubation time conditions, this difference is likely explained by the prolonged period between the two UV exposures and temperature differences (the biotin labeling/sorting process adds ~ 3 h to the incubation period, with ~ 2 h at 4 °C). These results indicate that the UVHR phenotype is passed onto subsequent cell generations, and not simply a product of the mother cell response to UV exposure.

We also considered the possibility that the UVHR phenotype might be genetic, potentially via mutations caused by the initial UV exposure. To explore this, we isolated random colonies produced from cultures that had been pre-exposed to 50 J/m^2^ UV, followed by a 4 h incubation prior to plating. The resultant colonies were re-streaked and incubated on fresh agar plates for ~ 72 h, to ensure that cells had grown far past the ~ 16–22-h period during which the UVHR phenotype is observed. These isolates were then used to perform a standard single UV exposure survival assay alongside isolates derived from unexposed control cultures. If the UVHR phenotype is due to mutational changes, it was predicted that a subset of the + UV isolates would display persistent UVHR.

The majority of + UV isolates exhibited survival frequencies that were comparable to those seen in the −UV isolates (Fig. 2b). A small fraction of + UV isolates displayed modestly increased survival relative to the controls, with a maximum of ~ tenfold increased survival. In contrast, a slightly larger fraction of + UV isolates displayed an ~ tenfold decrease in survival. The collective survival average of the + UV isolates was slightly lower than that of the −UV isolates, but this difference was not statistically significant. These results suggest that only a small percentage of cells acquire mutations that confer a hyper-resistance phenotype, and these effects are largely offset on the population level by mutations that cause hyper-sensitivity to UV. Furthermore, the degree of hyper-resistance observed in these select isolates is much smaller than that observed in the collective population (tenfold versus 200-fold). While we cannot fully rule out the possibility that there might be rare mutant isolates that exhibit high hyper-resistance that could explain the effect observed at the population level, such mutants would have to possess extremely high degrees of UV resistance to account for the aggregate UVHR phenotype. Thus, the collective results argue that the UVHR phenotype is not genetically based, but rather is a product of an epigenetic mechanism.

### Pre-exposure to UV radiation protects against subsequent UV damage

We subsequently addressed the question of the underlying molecular basis of the UVHR phenomenon. We initially considered the possibility that UVHR is a result of enhanced DNA repair during the secondary exposure to UV. UV radiation causes the formation of cyclopyrimidine dimers (CPDs), which are processed by a variety of DNA damage-induced repair processes, including nucleotide excision repair [10]. We speculated that repair processes might remain active after the repair of the initial damage, or that repair genes remain poised for hyper-activation during subsequent DNA damage events. If so, we anticipated that pre-exposed cells would be able to repair UV-induced CPDs more efficiently.

To test this hypothesis, samples of pre-exposed cells (and unexposed controls) were collected at various times following the secondary UV exposure. In these experiments, cells were either exposed (+ UV) or not exposed (−UV) to 50 J/m^2^ of UV radiation and incubated for 4 h (by which time CPDs were completely repaired). Both cultures were then exposed to 50 J/m^2^ of UV radiation. DNA was isolated from cells at various times before and after each exposure, and immunoblot assays were carried out using anti-CPD antibodies to quantify CPD levels (using equal quantities of DNA per slot blotted sample/culture condition). CPD levels were normalized to the damage levels detected immediately following the secondary exposure to determine the repair kinetics.

**Fig. 3:**
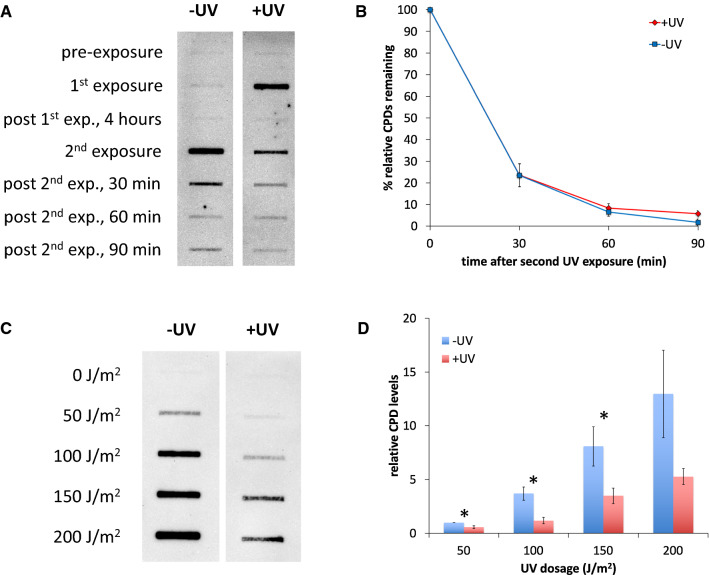
UV-exposed cells experience reduced DNA damage levels in response to subsequent UV exposure. Immunoblotting was done to assess UV-induced CPD formation and repair kinetics. Suspended yeast cells were UV exposed, as described in Fig. 1, and following indicated incubation periods, DNA was isolated from culture aliquots. Equal amounts of DNA were slot blotted and probed with anti-thymine dimer antibodies. Images were then evaluated by densitometry. Assays were done a minimum of three times, with mean ± 1 SE reported for each condition (*p < 0.01). **A**. UV repair kinetics. Log-phase cultures were initially exposed to UV at 50 J/m^2^ (+ UV), or mock exposed (−UV), followed by a 4 h incubation, and then a secondary UV exposure at 50 J/m^2^. Samples were collected at the indicated timepoints for DNA isolation and analysis. −UV and + UV images shown are from the same blot using identical exposure conditions. **B**. Densitometry analysis of the experiments presented in **A**, reported as the percentage of remaining CPD levels relative to the amount of CPDs present immediately after the second exposure. **C**. CPD levels acquired in pre-exposed (+ UV) and unexposed controls (−UV) during secondary UV exposures. Cultures were initially exposed to 50 J/m^2^, followed by a 4 h incubation, and then a secondary exposure at varying dosages (0–200 J/m^2^). Samples were collected immediately after the second exposure for DNA isolation and analysis. −UV and + UV images shown are from the same blot using identical exposure conditions. **D**. Densitometry analysis of the experiments presented in **C**, reported as CPD levels relative to the amount of CPDs present in the −UV/50 J/m^2^ samples

We found that the rate of removal of CPDs was indistinguishable between the −UV and + UV cultures (Fig. 3a, b). There was no significant difference in the fraction of relative CPDs remaining between the cells that were pre-exposed and those that were not over the subsequent 90 min evaluation period. These results indicate that altered repair kinetics are unlikely to be the cause of acquired UV hyper-resistance in yeast cells. However, we observed a pattern in the DNA samples collected immediately after the second UV exposure: the −UV cells displayed ~ 3.6-fold higher relative CPDs levels compared to the + UV cells (Fig. 3a, b). This observation suggests that the pre-exposed cells acquire less damage during the secondary UV exposure than the unexposed control cells.

To further substantiate this finding, we repeated these experiments by varying the secondary exposure dosage levels. These experiments showed similar results: in general, the −UV samples contained relative CPD levels ~ 2–3 times higher than the + UV samples (Fig. 3c, d). This difference was statistically significant at secondary exposure dosages of 50, 100, and 150 J/m^2^, and approached significance at the 200 J/m^2^ dosage. These results suggest that the UVHR phenotype is the result of a protective mechanism that is employed after the initial UV exposure to insulate against the formation of CPDs.

### Cell morphology changes following UV exposure may protect against subsequent exposure

Given the apparent DNA damage protection acquired during the initial UV exposures, we wished to gain further insights into the manner by which protection is achieved. We examined cells microscopically prior to and at various times after the initial UV exposure to determine if any obvious morphological changes occurred that correlated with the UVHR phenotype. Collected cells were stained with calcofluor white (CFW), a non-specific dye that binds to cellulose and chitin in yeast cell walls [17], and examined by fluorescent microscopy. Cells were digitally photographed and then analyzed to evaluate changes in CFW staining intensity and overall cell size.

**Fig. 4:**
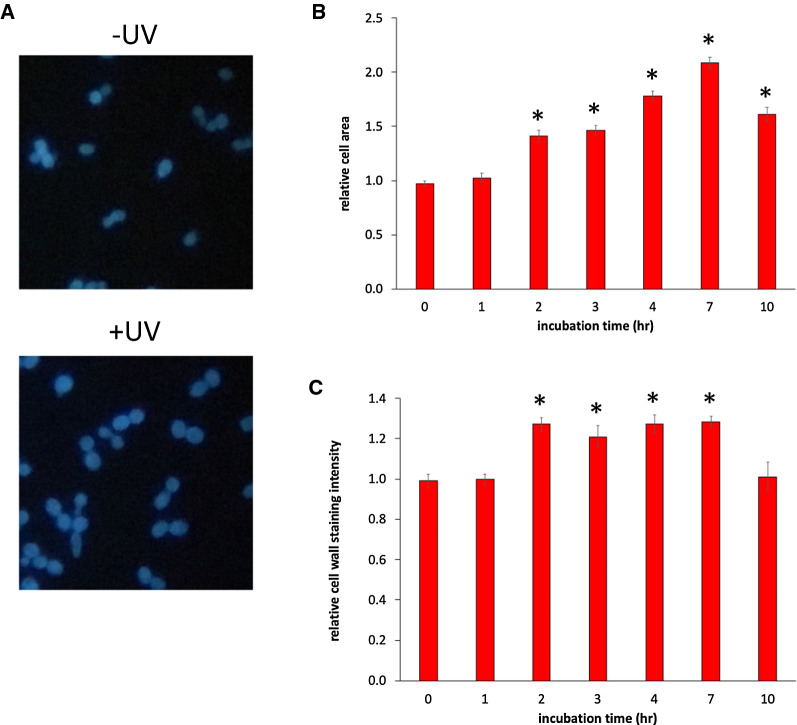
UV-exposed cells experience persistent increases in cell size and changes in cell wall composition. Log-phase suspensions were exposed to UV at 50 J/m^2^, or mock exposed, followed by incubation, as described in Fig. 1. Samples were collected from exposed cultures and unexposed controls at indicated timepoints and stained with calcofluor white. Cells were visualized by fluorescent microscopy and analyzed for cross-sectional cell area and fluorescent intensity. **A** Representative images from unexposed (−UV) and exposed (+ UV) cultures, following a 4 h incubation (400X magnification). **B** Cross-sectional cell area, reported as means ± 1 SE, normalized relative to the corresponding −UV cell size for each condition (*p < 0.01). **C** Calcofluor white (CFW) staining intensity, reported as means ± 1 SE, normalized relative to the corresponding −UV CFW staining intensity for each condition (*p < 0.01)

We found that UV exposed cells displayed an increase in size, beginning ~ 2 h after UV exposure, and persisting for at least 10 h, across multiple cell divisions (Fig. 4). The cross-sectional area of UV-exposed cells reached a maximum of a twofold increase relative to unexposed cells 7 h following UV exposure, remaining ~ 1.5-fold higher at the 10 h mark (Fig. 4b). In addition, UV exposed cells displayed ~ 1.3-fold increased CFW fluorescence beginning at the 2 h timepoint, persisting until the 7 h mark (Fig. 4c). Thus, these collective results indicate that UV exposure causes an increase in cell size and altered cell wall characteristics. The timeframe during which these changes persist roughly correlates with the duration of the UVHR phenotype.

In light of these observations, we executed a genetic screen to determine if UV-induced cell size changes might be responsible for the UVHR phenotype. We evaluated ~ 40 strains possessing knockouts of genes that have been previously demonstrated to have altered cell size, as reported in the Saccharomyces Genome Database [18]. We initially screened these strains for UV-induced size alterations, as described above, to identify mutants that displayed abnormal size changes in response to UV. About one quarter of these strains failed to enlarge in response to UV exposure (Additional file 1: Figure S2a; strains denoted with yellow boxes), and another quarter of these strains were found to be inherently large (in the absence of UV exposure), with minimal to no additional enlargement in response to UV (denoted with green boxes). To assess if the UV size response abnormalities had an impact on the UVHR phenotype, we subsequently evaluated cell survival of these two subgroups of mutant strains using the quantitative UV double-exposure assay described earlier (Additional file 1: Figure S2b). Regardless of the starting size of the cells or their lack of size changes in response to UV, we found that most of these mutant strains exhibited a normal UVHR phenotype. Only two strains (bem4 and fig 1) exhibited a partial reduction of the UVHR phenotype. These findings suggest that UV-induced cell size changes are not required for the UVHR phenotype.

**Fig. 5:**
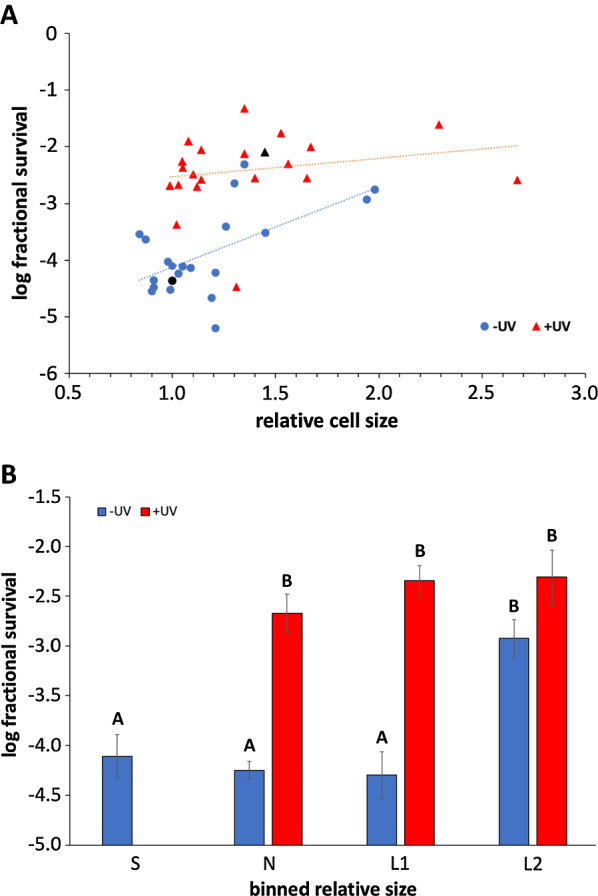
Relationship between yeast cell size and UV resistance. **A** Strain cell size was correlated with UV survival, based on data reported in Additional file 1: Figure S2a, b. Survival frequencies are log-converted values, accompanied by best fit lines for the pre-exposed (+ UV; *R*^2^ = 0.05; *p* = 0.34) and unexposed conditions (−UV; *R*^2^ = 0.35; *p* = 0.003). Darkened data points indicate the wildtype strain. **B** Log-converted UV survival frequencies in pre-exposed (+ UV) and unexposed (−UV) strains, binned based on relative cell size ranges: S (smaller than −UV wildtype; 0.85–0.95 relative size); N (normal; comparable to −UV wildtype; 0.95–1.05); L1 (modestly larger than −UV wildtype; 1.05–1.25); L2 (much larger than −UV wildtype; comparable to + UV wildtype;  > 1.25). The lack of a bar for “S + UV” indicates that no strains were in the indicated size range. Values are means ± 1 SE. For data sets not sharing a letter on the graph, *p* < 0.01 (except S- versus L2-, *p* = 0.02)

However, we did note varying degrees of general survival in response to UV exposure across these strains. When examining UV survival in the absence of pre-exposure, we observed a modest, statistically significant linear relationship relative to cell size, with larger cells exhibiting higher survival frequencies (Fig. 5a). Interestingly, we found that increased survival is specifically observed in strains that are  > 1.25-fold larger compared to unexposed wildtype cells, corresponding roughly to the size of UV-exposed wildtype cells (binned group L2 in Fig. 5b). The survival frequency observed in these strains is comparable to that seen in UV-exposed wildtype cells. These results indicate that increased cell size serves as a protective mechanism against UV. Thus, while the UV-induced cell size increase is not explicitly required for UVHR, this induced morphological change may be a contributing component to the phenotype.

To evaluate the role of UV-induced cell wall changes in the context of the UVHR phenotype, we similarly executed a genetic screen to identify genes required for cell wall synthesis/assembly/maintenance that might be required for UVHR. We executed a qualitative UV double-exposure assay on ~ 50 yeast strains that possessed knockouts of non-essential cell wall-related genes. In general, we found that all strains exhibited largely normal hyper-resistance to UV following an initial 50 J/m^2^ pre-exposure, with only minor differences observed between the mutant and wildtype strains (Additional file 1: Figure S3). Thus, while there is a correlation between the UVHR phenotype and changes in cell wall morphology, we cannot definitively demonstrate that UVHR is caused by cell wall changes.

### Specific histone post-translational modifications are important for UVHR

Our final goal was to identify the inheritable epigenetic factors that propagate the UVHR phenotype. As addressed earlier, histone modifications have been identified as potential epigenetic mediators, and may be inherited through cell division. To pinpoint histone modifications that contribute to the UVHR phenotype, yeast strains lacking genes that encode for specific histone modification enzymes (or genes that regulate these modifications) were evaluated for UVHR phenotype using the qualitative UV double-exposure assay described above. Cells either received a primary 50 J/m^2^ exposure or were left unexposed, followed by a 4 h incubation period and subsequent UV exposures at higher dosages. Strains that displayed reduced UVHR were subsequently evaluated via a quantitative UV double-exposure assay to characterize the impact of the specific gene knockouts more precisely with respect to the UVHR phenotype.

**Fig. 6:**
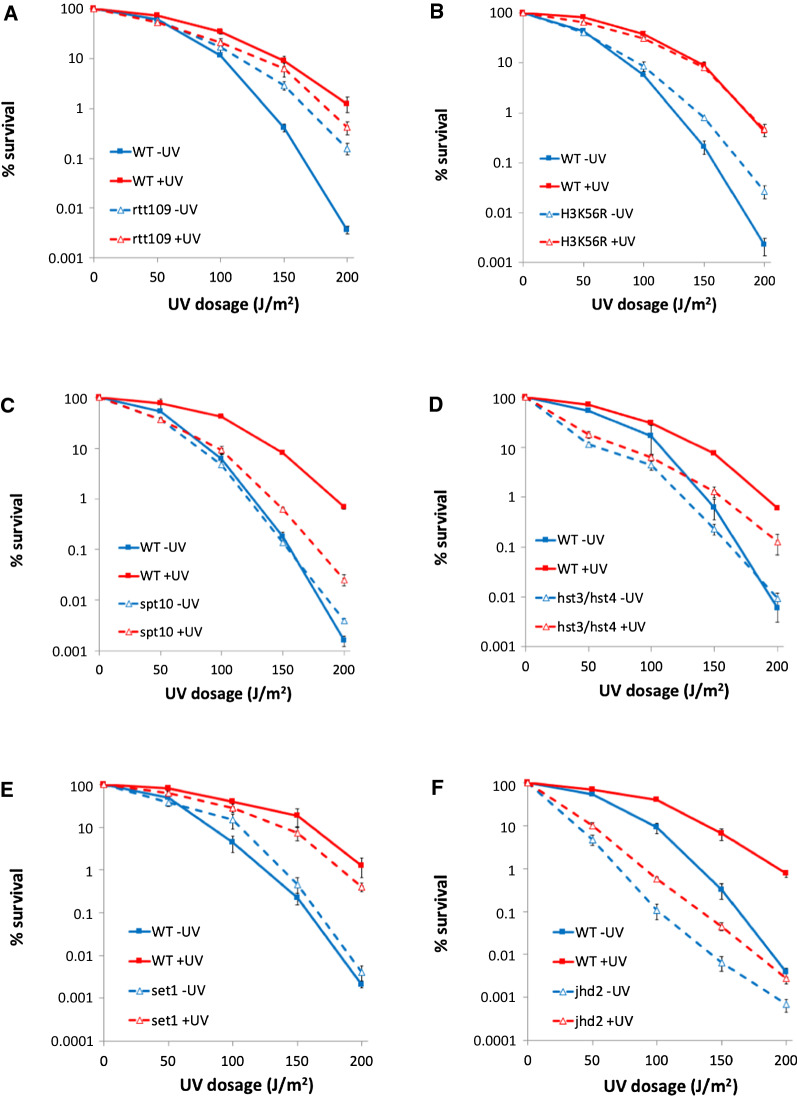
UV double-exposure survival analysis of histone modifier mutant strains. Assays were done as described in Fig. 1. **A** Histone H3K56 acetyltransferase rtt109 mutant. **B** Histone H3K56R mutant. **C** Histone H3K56 acetylation regulator spt10 mutant. **D** Histone H3K56 deacetylase hst3/hst4 double-mutant. **E** Histone H3K4 methyltransferase set1 mutant. **F** Histone H3K4 demethylase jhd2 mutant

While most of the mutant strains exhibited only very slight or no impact on UVHR (Additional file 1: Figures S4 and S5), several mutant strains were identified that displayed notable alterations to the UVHR phenotype (Fig. 6), focusing our attention on H3K56 acetylation (H3K56ac) and H3K4 methylation (H3K4me). With regards to H3K56ac, we observed that deletion of RTT109, which encodes for histone H3K56 acetyltransferase Rtt109 [19], resulted in an increase in resistance to the initial UV exposure, while concurrently eliminating subsequent hyper-resistance conferred by pre-exposure (Fig. 6a). To confirm the role of this modification in UVHR, we examined the response in the unacetylatable histone H3K56R mutant. Comparable to the rtt109 mutant, the H3K56R mutant displayed increased general resistance to UV relative to the wildtype strain in the absence of pre-exposure, albeit to a lower degree than the rtt109 strain (Fig. 6b), accompanied by a modest ~ 20-fold increased resistance in response to UV pre-exposure. Furthermore, we found that deletion of SPT10, which is required for cell cycle-specific acetylation of H3K56 of select histone genes [20, 21], resulted in a substantial reduction of the UVHR phenotype (Fig. 6c). Finally, while deletion of the individual HST3 and HST4 genes, which encode for redundant H3K56 deacetylases [22], had no impact on UVHR (Additional file 1: Figure S5), deletion of both genes resulted in a partial reduction in the UVHR phenotype (Fig. 6d). These collective results indicate that H3K56ac is important for the UVHR response.

**Fig. 7:**
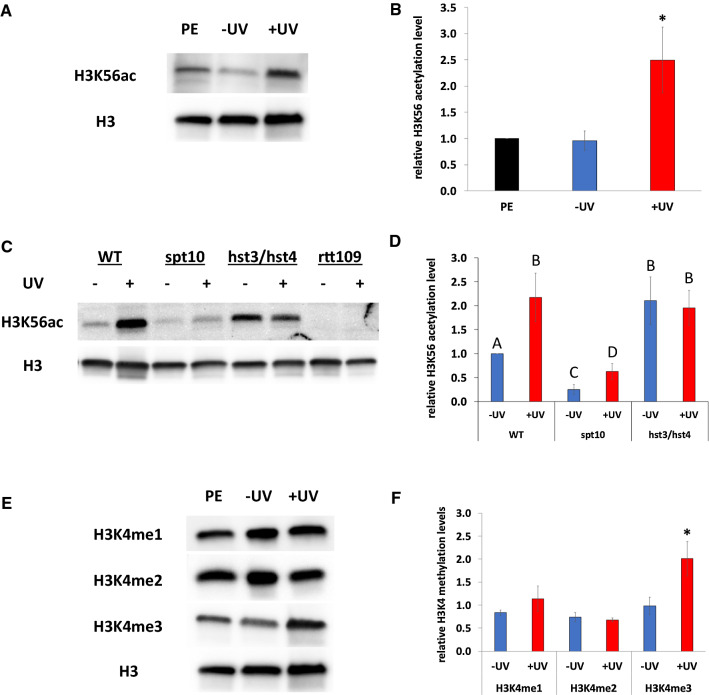
Histone H3K4 methylation (H3K4me) and H3K56 acetylation (H3K56ac) levels in UV-exposed cells. Log-phase cultures were exposed to UV at 50 J/m^2^, followed by a 4 h incubation. Samples were then collected, and isolated proteins were analyzed by western blot with histone modification-specific antibodies. Blots were subsequently analyzed by densitometry. Values were initially normalized relative to general H3 values to correct for histone level variation, and then subsequently normalized relative to the pre-exposure values for each modification (except panel D; normalized to unexposed wildtype cultures). Values are reported as means ± 1 SE reported for each condition. + UV, exposed cells; −UV, unexposed controls; PE, pre-exposure cells. **A**, **B**. H3K56 acetylation in wildtype cells. *p < 0.01. **C**, **D**. H3K56 acetylation in wildtype (WT), spt10, hst3/hst4, and rtt109 mutant strains. For data sets not sharing a letter on the graph, *p* < 0.01. **E**, **F**. Histone H3K4 methylation levels in wildtype cells. H3K4me1, monomethylation; H3K4me2, dimethylation; H3K4me3, trimethylation. *p < 0.01

To evaluate how H3K56ac is regulated in response to UV exposure, we employed western blotting to measure acetylation levels in UV-exposed cells after the 4 h incubation period. We found that UV-exposed cells experienced a ~ 2.5-fold increase in H3K56ac levels relative to pre-exposure levels (as well as compared to unexposed cells after the same incubation period; Fig. 7a, b). As expected, no acetylation was observed in the rtt109 strain in response to UV, indicating that Rtt109 is required for UV-induced acetylation (Fig. 7c, d). In the spt10 strain, H3K56ac levels were reduced to ~ 20% of wildtype levels in absence of UV. UV exposure caused a corresponding threefold increase in acetylation in the spt10 strain, but overall H3K56ac levels remained proportionately reduced relative to UV-exposed wildtype levels. Conversely, H3K56ac was elevated in the hst3/hst4 strain, regardless of UV exposure conditions, with levels comparable to those observed in the UV-exposed wildtype cells. These collective results suggest that increased H3K56 acetylation by Rtt109, regulated by Spt10, is an important feature of the UVHR phenotype.

We similarly found that deletion of genes involved in H3K4me influences UVHR. Deletion of SET1, which encodes for the H3K4 methyltransferase [23], had no impact on UVHR (Fig. 6e). However, deletion of JHD2, which encodes for an H3K4 demethylase [23], resulted in decreased general resistance to UV, while also reducing the UVHR response (Fig. 6f). These results indicate that while H3K4me is not required for UVHR, regulation of this modification via the Jhd2 demethylase is important for this phenotype. We subsequently examined UV-induced changes in H3K4me by western blot. We found that methylation levels at this site increased in response to UV during the subsequent post-exposure incubation period (Fig. 7e, f). However, the response was uniquely observed with respect to trimethylation, which increased ~ twofold, with no significant changes observed with respect to mono- or di-methylation. The collective results indicate that UV-induced changes in H3K4me3 levels are important for UVHR.

## Discussion

Our findings point to the existence of a UV hyper-resistance phenotype, in which cells that have experienced modest degrees of DNA damage in response to an initial UV exposure are afforded a protective mechanism that notably increases survival in response to a subsequent UV exposure. We find that the increased survival is correlated with reduced amounts of DNA damage generated during the secondary UV exposure, suggesting that pre-exposed cells implement a form of protection to insulate against future UV induced damage. Furthermore, we demonstrate that this is an inheritable phenotype in a manner that appears to be epigenetic in nature. The phenotype persists for ~ 7–8 mitotic generations, before gradually diminishing. To the best of our knowledge, this is the first reporting of an inheritable epigenetic process in the context of DNA damage response. The term “epigenetic” has taken on a variety of meanings in recent years, ranging from the description of molecular processes involving “epigenetic marks”, inheritable gene expression patterns, to inheritable phenotypes in the absence of genetic change [1]. Our observations regarding the UVHR phenotype fall into the latter “inheritable phenotype” category, although the underlying processes likely involve epigenetic marks and inheritable gene expression patterns. As noted earlier, several prior studies have hinted at cellular responses that occur in the context of multiple exposures to UV, but ours is the first to demonstrate that this phenomenon is passed on generationally, in the absence of underlying genetic change.

This is certainly not the first example of multigenerational epigenetic inheritance, and the process that we have characterized shares similarities with other such examples. Numerous cases have been reported in which cells undergo phenotypic change and/or changes in gene expression in response to environmental stimuli (including galactose induction, heat shock, interferon induction, inositol starvation, and tissue damage) that are epigenetically inherited [2, 24]. One common characteristic across these examples is the persistence of the phenotypic/gene expression change through multiple rounds of mitotic or meiotic reproduction, followed by a gradual diminishing of the phenotype over time/generations. These patterns are the hallmark of epigenetic inheritance, highlighting the capacity for phenotypic plasticity in response to environmental cues, providing a means for cells/organisms to remain responsive to future triggers. At the same time, the reversible nature of epigenetic marks enables cells to restore phenotypes and/or reset gene expression patterns once sufficient time has passed without subsequent exposures to the initiating signal. Such responses are undoubtedly valuable in the context of DNA damage response, particularly given the regularity with which organisms experience exposures to environmental mutagens like UV.

We have identified potential candidates for UVHR-associated epigenetic marks in the form of two key histone modifications: H3K56 acetylation and H3K4 methylation. Loss of the former modification leads to a reduction of the UVHR phenotype, while also causing an overall increase in the general resistance to UV, adding a function to an already extensive list of roles in the response to DNA damage and the management of genome stability by this modification [19, 25, 26]. We also find that hyperacetylation at this site, as evaluated in the hst3/hst4 double mutant strain, also weakens UVHR. Although we find that overall H3K56ac levels rise in response to UV, the collective results indicate that it is not simply the presence or absence of H3K56ac that is important for UVHR, but rather the regulation of this modification in response to UV damage, potentially in a locus-specific manner. This point is suggested by the severe reduction of UVHR in the spt10 mutant strain. SPT10 is required for H3K56 acetylation at several histone gene promoters [20, 21], serving to regulate cell cycle-related histone expression. These connections suggest that histone dosage may play a role in the UVHR response. Prior studies have implicated changes in histone dosage in the response to DNA damage [27–29], and thus additional studies are merited to further explore this potential mechanism.

Histone H3K4 methylation is not required for UVHR, but the inability to remove this modification, as evaluated in strains lacking the H3K4 demethylase JHD2, results in a notable reduction of the phenotype. Paradoxically, we find that overall H3K4me3 levels rise in response to UV, suggesting that the role of demethylation in UVHR may be locus specific. A possible role for this modification is suggested by prior work on yeast sporulation, in which H3K4me3 is specifically demethylated at intergenic loci as a means of abrogating spurious intergenic transcription [30]. It is conceivable that a comparable role for H3K4me3 may serve to support UV-induced gene expression patterns that are important for UVHR.

It is likely that other histone modifications play additional contributing roles to the UVHR phenotype. A subset of the histone modifier mutants that we examined display modest reductions in UVHR (Additional file 1: Figures S4 and S5), and even amongst the mutants that exhibit strong phenotypic effects, none cause a complete loss of UVHR, arguing that multiple modifications play redundant and complementary roles in the process. This is specifically supported by modest phenotypic differences between the rtt109 and H3K56R mutants. Both mutations cause a general increase in resistance to UV, but the effect is more pronounced in the rtt109 strain (Fig. 6A, B), suggesting that Rtt109 may have additional modification targets that contribute to UVHR. While the primary role for Rtt109 is acetylation of H3K56, it has also been shown to secondarily contribute to H3K4 and H3K27 acetylation [31, 32]. While none of the other histone modifier mutants that we examined provide compelling evidence to suggest a role for either of these modifications in UVHR, further evaluation is merited.

The underlying protective mechanism responsible for UVHR remains to be fully characterized, but we have identified some potential clues to this process. Specifically, we have established a strong correlation between the UVHR phenotype and UV-induced changes in cell size and cell wall characteristics. In particular, we have observed that increased cell size is associated with general resistance to UV, particularly amongst strains whose size exceeds the 1.25-fold relative threshold, suggesting that UV-induced size increase contributes to the UV-induced protection against DNA damage. UV-induced size increase explains a partial degree of the overall UVHR phenotype, but we have been unable to identify mutants that demonstrate a definitive cause–effect relationship between either UV-induced size and/or cell wall changes and the UVHR phenotype. Our findings do not necessarily rule out either of these responses as part of UVHR, and it is plausible that the overall UVHR response is comprised of multifaceted, redundant processes, thus preventing single-gene knockouts from causing disruptions to the UVHR process. Additional studies exploring this phenotype in strains concurrently defective for UV-induced cell size and cell wall changes are needed. Further clues about the nature of UVHR are suggested by the preliminary analysis of the bem4 and fig 1 mutants, both of which display partial UVHR defects (Additional file 1: Figure S2b). BEM4 is involved in bud emergence and cell polarity [33], while FIG1  participates in cell fusion during mating [34]. Exploration of these processes in the context of UVHR is merited.

An additional factor to consider with respect to the nature of the UVHR phenotype is the potential role of cell cycle regulation. A prior study demonstrated that UV sensitivity in yeast varies during the cell cycle, with maximal resistance observed during G2 [35]. The underlying cause for this effect is not understood, but it is possible that this observation may relate to the relationship between UV resistance and cell size reported here. Yeast cell size is regulated during the cell cycle, mediated by various processes that primarily take place during G1 [36], with recent studies pointing to additional regulation during G2 [37]. It is conceivable that exposure to UV triggers changes in cell cycle progression after the resolution of DNA damage checkpoint activation, leading to enrichment of UV hyper-resistant G2 cells in the growing culture population. Furthermore, it has been shown that prolonging the cell cycle leads to an increase in yeast cell size [38], which may enhance the UVHR phenotype. The epigenetic nature of the UVHR phenotype suggests that the putative altered cell cycle distribution and associated cell size changes subsequently propagate over multiple cell generations to provide inheritable protection against recurrent UV exposure. Additional studies to explore the relationship between UVHR and the yeast cell cycle may provide an enhanced understanding of the underlying process.

While additional work is needed to integrate the observations presented above into a cohesive model, we are inspired to speculate that the observed UV induced histone modification changes lead to altered gene expression patterns. This in turn gives rise to the observed UV protective cellular changes, including increased cell size and cell wall structure (amongst other yet-to-be identified features). These protective changes abate the degree of DNA damage in response to subsequent UV exposures, thus increasing the likelihood of cellular survival. Preservation of UV induced histone modification and gene expression changes through mitosis would serve to epigenetically propagate the UVHR phenotype. The proposed connection between histone modifications, cellular changes, and UV resistance is particularly exemplified by the rtt109 mutant strain, in which the lack of H3K56ac is associated with both increased cell size and resistance to UV. Future studies to examine UV-induced genome-level expression and the histone modification landscape will prove insightful to evaluate this model, as well as to identify other cellular processes that contribute to the protective aspects of UVHR.

## Conclusions

We demonstrate here the existence of an epigenetically inherited DNA damage response process that protects yeast cells against repeated exposures to UV radiation. Pre-exposed cells experience reduced levels of UV-induced DNA damage, resulting in substantially increased survival frequencies. While the underlying mechanism is not fully understood, UV-induced increase in cell size and/or change in cell wall composition may be contributing factors. In addition, we have identified two histone post-translational modifications, H3K4 methylation and H3K56 acetylation, that are important for this response, potentially serving as the epigenetically inherited instructions that mediate this process. We speculate that UV induced histone modification changes influence expression of genes that promote the cellular changes required for protection against sequential UV exposures.

## Materials and methods

### Yeast strains

*S. cerevisiae* wildtype strain BY4741 and isogenic knockout strains were obtained from the Saccharomyces Genome Deletion Project (GE Dharmacon) [39]. Additional strains were obtained as follows (all isogenic to BY4741): set1 knockout strain SDBY1210 [40]; hst3/hst4 double knockout strain MT15 [41]; and histone H3K56R strain YVY78 [42].

### UV double-exposure quantitative survival assays

UV double-exposure survival assays were adapted from a previously established UV exposure methodology [43]. Yeast strains were grown overnight to mid-log phase in YEPD broth and re-suspended in sterile water. Half of the cell suspension was exposed to the appropriate dosage of UV radiation (typically 50 J/m^2^), in 60 mm Petri dishes on a rotating platform, at 254 nm using a Philips 30 W G30T8 UV lamp (luminosity measured by a UVX radiometer; UVP, Inc.). The other half of the original suspension was left unexposed. Each suspension was added to appropriate volumes of 10X YEPD media and incubated at 30 °C with shaking for the indicated time. For assays that involved extended incubations (> 7 h), the post-exposure cultures were serially diluted prior to incubation to ensure that cultures remained in log phase throughout the incubation period. Following incubation, cultures were serially diluted and plated onto YEPD agar in duplicate. Plates then received a secondary UV exposure (typically 0, 50, 100, 150, and/or 200 J/m^2^), followed by incubation in the dark at 30 °C for 6 days. Colonies on each plate were counted, and average percent survival relative to the unexposed control plates was calculated for each strain at each secondary UV dosage. Reported values are means ± 1 SE of at least three independent experiments. Log-converted survival frequencies were statistically analyzed by ANOVA, accompanied by a Tukey HSD post hoc test (JMP Pro version 15, SAS Institute, Inc.).

### Biotin-labeling cell separation

To remove mother cells in the above-described quantitative UV double exposure assays, UV-exposed cell populations (and the corresponding unexposed control cultures) were labeled with biotin immediately following the first UV exposure and removed via streptavidin-linked magnetic beads [16, 44]. Cultures were centrifuged immediately after UV exposure and resuspended in 1 ml of PBS. EZ-link Sulfo-NHS-LC-Biotin (ThermoFisher) was added to each suspension at 4 ug/ul and incubated on a nutator at room temperature for 15 min. Samples were subsequently centrifuged and washed three times with PBS, and then resuspended in YEPD broth for the post-UV exposure incubation, as described above. Following the 4 h incubation, samples were centrifuged, washed, and resuspended in 1 ml of PBS and 100 μl of pre-washed MagnaBind Streptavidin beads (ThermoFisher). Samples were incubated on a nutator for 2 h, followed by incubation in a magnetic microcentrifuge rack for 20 min (both at 4 °C). Supernatant containing daughter cells was collected, and cell density was calculated with a hemacytometer. Samples were diluted to 3 × 10^7^ cells/ml, and then serially diluted 1:10 for plating and UV exposure. Data were processed and analyzed as described in the quantitative double-exposure method above.

### UV double-exposure qualitative survival assays

Yeast strains were inoculated in 100 ul of YEPD broth in a 96 well plate (typically 7 mutant strains plus wildtype strain BY4741). Cultures were incubated overnight at 30 °C on a 96 well plate shaker at 1000 rpm. The next day, cultures were serially diluted 1:10 and incubated while shaking for an additional 18 h. Plates were subsequently centrifuged to pellet cells; for each strain, the most dilute culture that produced a visible pellet was selected to ensure that each culture was in mid-log phase (while also having sufficient cells to complete the analysis). The selected pellets were washed and resuspended in sterile water. The suspensions were divided and transferred to two new 96 well plates; one plate was subsequently exposed to 50 J/m^2^ of UV radiation (254 nm, Philips 30 W G30T8 UV lamp). Appropriate volumes of 10X YEPD were added to all suspensions. Plates were covered to avoid light exposure and were incubated at 30 °C on a 96 well plate shaker at 1000 rpm for 4 h.

After incubation, cells were washed and resuspended in 100 μl (−UV samples) or 35 μl (+ UV samples) of sterile water, to compensate for survival differences. Cultures from both 96 well plates were then serially diluted 1:10 to 10^−5^, followed by spot plating onto YEPD agar plates in duplicate. Pairs of plates were exposed to a second UV dosage of 0, 150, or 200 J/m^2^, and then incubated at 30 °C for ~ 4 days. Plates were qualitatively analyzed by comparison of each strain’s response to the secondary dosage of UV with or without the primary exposure event. Each strain was evaluated at least three times.

### DNA damage immunoblotting

UV-induced DNA damage was evaluated by immunoblotting, using a protocol adapted from McCready [45]. Cultures were grown overnight to mid-log phase at 30 °C. Appropriate volumes of washed cells were placed in a 150 mm petri dish on a rotating platform and exposed to 50 J/m^2^ UV as described above. The exposed cells and unexposed control cells were transferred to flasks containing an appropriate volume of 10X YEPD broth and incubated shaking at 30 °C for the indicated time periods. For double-exposure assays, cultures were washed, re-exposed to UV at varying dosages, and incubated in YEPD broth. Culture samples were collected at indicated times and mixed with an equal volume of 100% ethanol on ice to fix the cells. Cells were centrifuged, washed with sterile water, recentrifuged, and stored at −80 °C.

DNA was isolated from collected samples using the DNeasy Blood & Tissue Kit (Qiagen). Cell pellets were resuspended in 600 µl sorbitol buffer (1 M sorbitol, 100 mM EDTA, with 0.59 µl β-mercaptoethanol per mL of buffer) containing 0.5 mg zymolyase per ml (Amsbio), incubated at 30 °C for 30 min on a nutator. Cells were subsequently mixed with an equal volume of AL buffer from the kit and examined microscopically to ensure that at least 95% of cells were spheroplasted. Spheroplasts were centrifuged for 10 min at 300 × g, and the pellets were then processed as per kit instructions.

To quantify and normalize DNA yields between the preps, DNA samples were diluted 1:5 in TE and mixed with 2 µl ethidium bromide (2 µg/ml; 10 µl total volume). Aliquots were spotted onto a non-UV absorbing gel casting tray, along with known quantities of a control DNA sample (High Mass DNA Ladder, ThermoFisher). Samples were visualized by UV transillumination, and an image was captured using a Fluor Chem HD2 imaging system (Alpha Innotech; 3 s exposure). Densitometry of the image was done using FluorChem2 software to determine relative DNA abundance, which was subsequently used to adjust concentrations of DNA samples. Adjusted samples were retested to ensure equivalent concentrations.

Appropriate volumes of DNA were combined with TE to a total volume of 125 µl, mixed with 13.75 µl 1 N NaOH, and incubated at room temperature for 5 min. To neutralize the solution, 111.25 µl prechilled KOAc/5 M acetic acid solution was added to each sample. Samples were then transferred to a prechilled 96-well plate and serially diluted 1:4 in 1 M NH_4_OAc. Sample dilutions were then transferred onto a Hybond-N + membrane (GE Healthcare) using a BioDot SF Microfiltration Apparatus (BioRad), followed by a wash with 0.4 N NaOH. The membrane was rinsed in 2X SSC and allowed to air dry, followed by baking at 80 °C for 30 min.

Membranes were blocked with 5% milk in TBS-Tween for 1 h at room temperature on a rocker platform. The membrane was then incubated with anti-thymine dimer antibody (Abcam #ab10347, 1:2000 in 0.1% Tween + 0.5% milk + 0.02% NaN_3_) overnight at 4 °C. The membrane was subsequently washed with 0.1% TBS-Tween, and then incubated with goat anti-mouse IgG-HRP antibody (Abcam #ab205719; 1:3000 in TBS-Tween + 3% milk) at room temperature for 1 h. After incubation, the membrane was washed with 0.1% TBS-Tween, and then incubated with ECL2 chemiluminescence reagents (ThermoFisher). The blot was imaged using the Fluor Chem HD2 imaging system (Alpha Innotech). Densitometry analysis of the captured image was conducted using ImageJ software [46], using dilutions whose band intensities were within an appropriate linear range. Assays were repeated 5–7 times; data reported represents means ± 1 SE. Data were statistically analyzed by ANOVA, accompanied by a Tukey HSD post hoc test.

### Cell size and cell wall assays

Yeast cultures were grown overnight until they reached mid-log phase. Cultures were washed and resuspended in sterile water, and then exposed to UV (50 J/m^2^) in a 60 mm Petri dish on a rotating platform, as described above. UV exposed cultures and corresponding unexposed controls were subsequently supplemented with 10X YEPD and incubated shaking at 30 °C.

At desired timepoints, samples were collected, washed, and resuspended in sterile phosphate buffered saline. Equal amounts of the resuspended culture, calcofluor white (Sigma Aldrich), 10% KOH, and SlowFade Gold Antifade Reagent (ThermoFisher) were combined on a microscope slide and incubated for 1 min. Cells were visualized at 400X on a Zeiss Axiovert 40 CFL fluorescent microscope, and images were captured with either a Lecia DFC3000-G or an Accu-Scope Excelis HD camera. Images were imported into ImageJ, and the intensity and cross-sectional area of at least 20 cells per sample/timepoint was measured and used to calculate mean relative cross-sectional area and intensity (normalized to the unexposed wildtype controls). Experiments were repeated 3–5 times, and data reported are averages of the individual experimental trials. Data were statistically analyzed by ANOVA, accompanied by a Tukey HSD post hoc test.

### UV exposure western blots

Histone modification levels in UV exposed cells were measured by western blotting. UV exposure of yeast cultures [47] and preparation of whole cell extracts [48] was done as previously described. Western blotting was done as previously described [47], using primary anti-H3 (Abcam #1791, 1:7500), anti-H3K56ac (Millipore #07-677-I, ~ 1:1000), anti-H3K4me1 (Active Motif #39,297; ~ 1:50,000), anti-H3K4me2 (Millipore #07–030; ~ 1:100,000), anti-H3K4me3 (Active Motif #39,159; ~ 1:100,000), and secondary goat anti-rabbit IgG-HRP (Millipore #12–348, 1:3000) antibodies. UV culture exposures/protein isolations were done 3–5 times, and each whole cell extract set was evaluated by western blot 2–3 times. Densitometry was done using ImageJ. Data reported are means of the collective assays, which were statistically evaluated by Kruskal–Wallis one-way nonparametric analysis.

## Supplementary Information

**Additional file 1. **Figures S1-S5

## Data Availability

All relevant data are within the paper and its additional files. The data used to support the findings of this study are available upon reasonable request.

## Abbreviations

UV radiation: Ultraviolet radiation
UVHR: UV hyper-resistance
H3K4me: Histone H3 lysine-4 methylation
H3K56ac: Histone H3 lysine-56 acetylation
