# Supplementary material for: An epigenetically inherited UV hyper-resistance phenotype in Saccharomyces cerevisiae
Source: Epigenetics Chromatin. 2022 Aug 20;15:31. doi: 10.1186/s13072-022-00464-5 (PMC9392361; doi:10.1186/s13072-022-00464-5)
Supplement: Supplementary file 1 — Additional file 1. Figures S1-S5 [file 13072_2022_464_MOESM1_ESM.pdf]

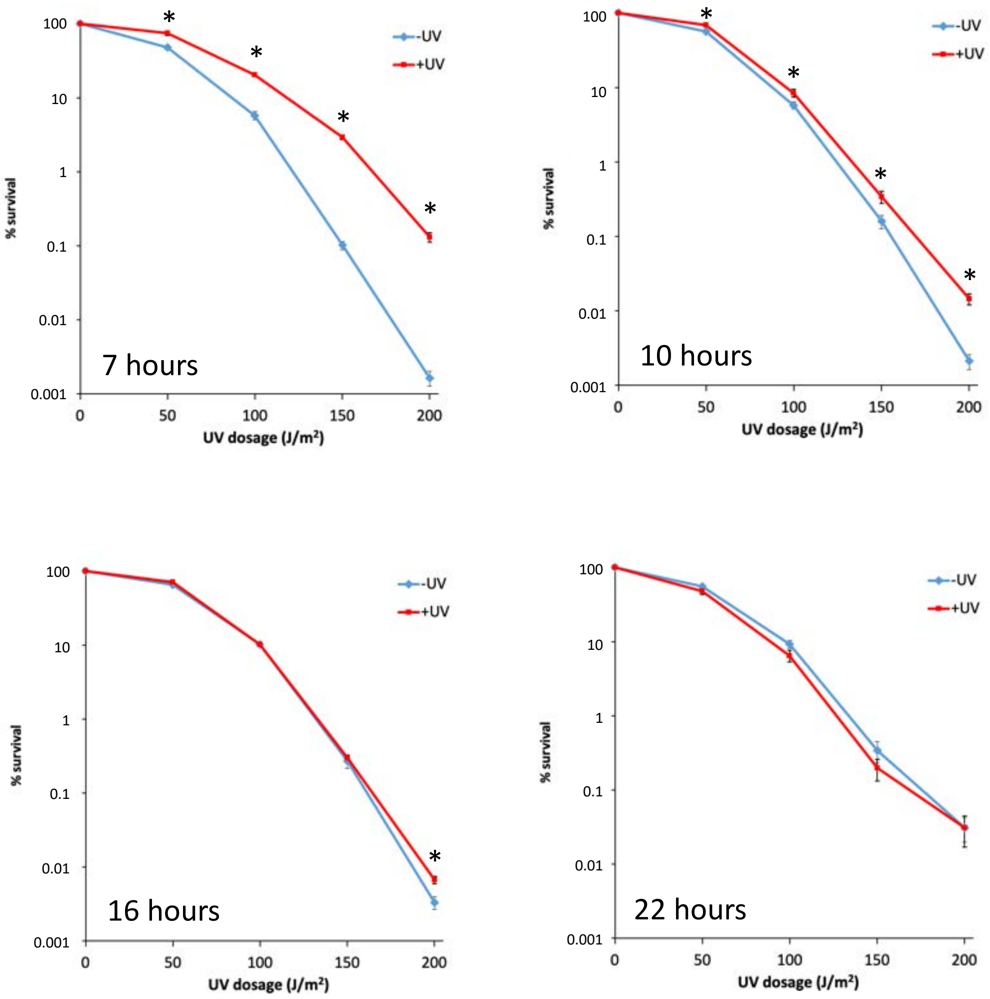

Figure S1. Quantitative UV double-exposure survival assays. Executed as described in Figure 1, except with varying incubation times, as indicated on graphs. *, $p < 0.01$.

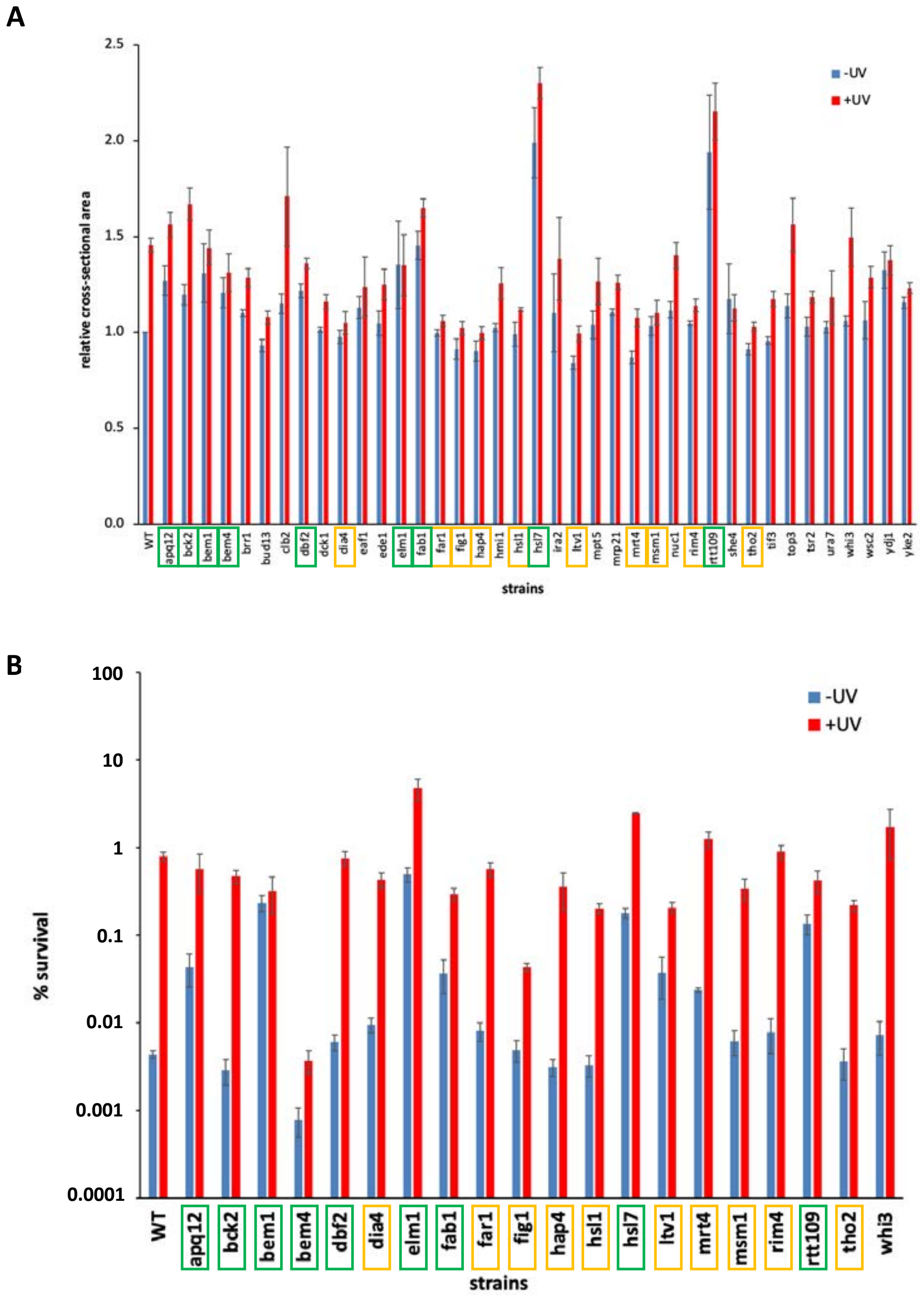

Figure S2. Screening of yeast mutants for UV-induced cell size defects. A. Yeast strains lacking genes that have been previously implicated with cell size regulation were evaluated for UV-induced size changes. Gene names listed on X axis. Cells were exposed to UV (+UV; 50 J/m^2), along with unexposed controls (-UV). After a 4-hour incubation, cells were examined by microscopy and measured for cross-sectional area, as described in Figure 4. Values were normalized relative to the unexposed wildtype (WT) size, reported as means ± 1 SE of at least three replicate assays. Yellow boxes designate strains whose size did not change in response to UV exposure; green boxes designate strains that were inherently larger (and exhibited little to no change in size in response to UV). B. Mutant strains identified that exhibited cell size anomalies were exposed to an initial dosage of UV (50 J/m^2) or unexposed, followed by a 4-hour incubation, and then a subsequent UV exposure (200 J/m^2). Whi3 was also included, based on its established role in cell size regulation. Survival frequencies were calculated as described in Figure 1. Strains were tested 2-3 times, and data are reported as means ± 1 SE.

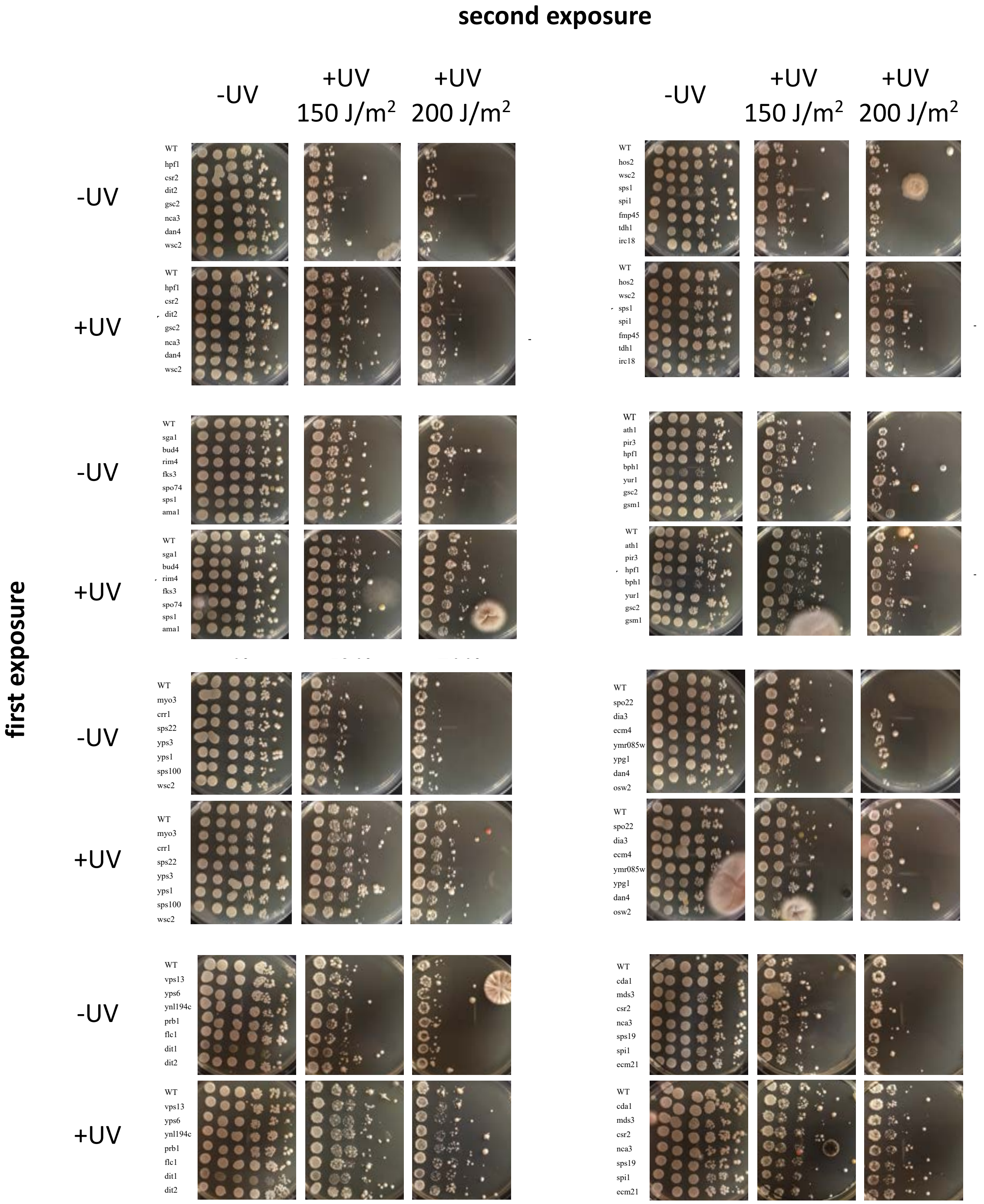

Figure S3. Yeast strains lacking specific cell wall-related genes screened for altered UVHR phenotypes by a qualitative double-exposure assay. UV exposures were done as described in Figure 1, except that culture growth and initial UV exposures (50 J/m^2; indicated with +UV on the left side) were done in 96-well plates. Following a 4-hour incubation, 10-fold serially diluted cultures were spot-plated on YEPD and then exposed to a secondary UV dosage (indicated at top; 0, 150, or 200 J/m^2). Plates were incubated for ~5 days, and then photographed using a digital camera. Each strain was tested a minimum of three times. A representative data set is shown, with gene knockout names indicated on the left.

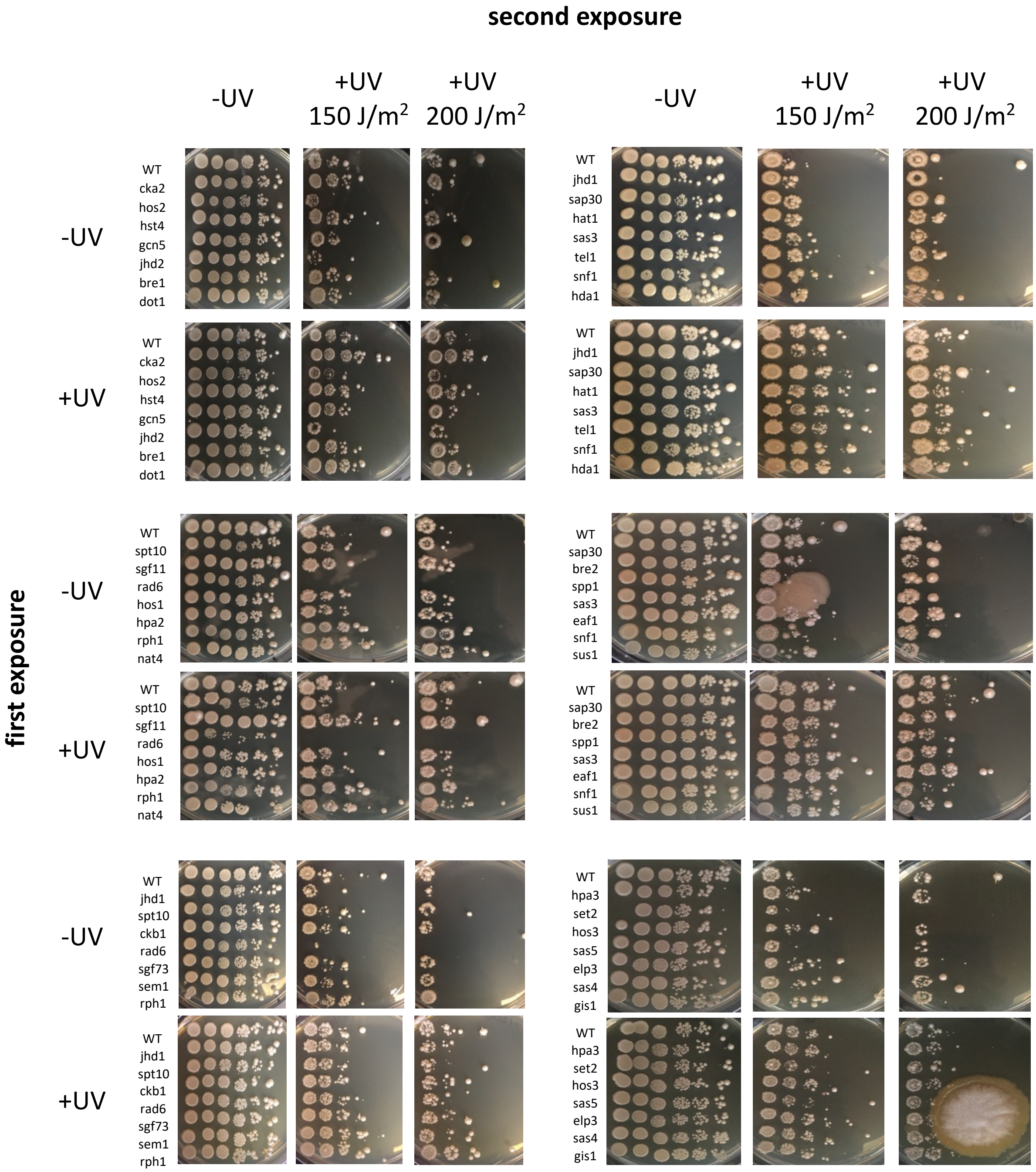

Figure S4. Yeast strains lacking specific histone modifier genes screened for altered UVHR phenotypes via a qualitative double-exposure assay, as described in supplemental Figure S3. A representative data set is shown, with gene knockout names indicated on the left.

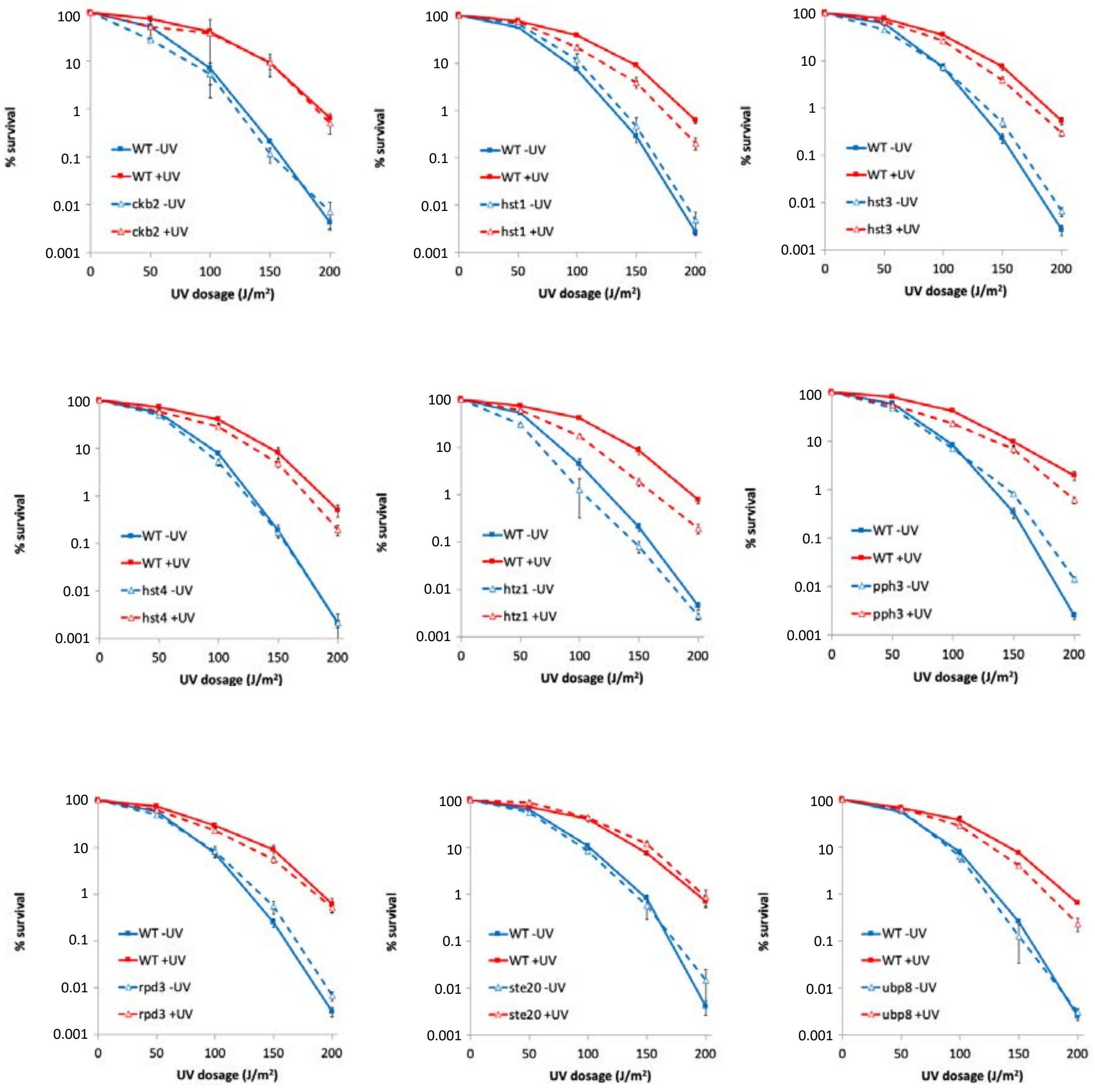

Figure S5. UV double-exposure quantitative survival assays on histone modifier mutants. Executed as described in Figure 1. Gene knockout names indicated on each graph.
